# Preparation and Properties of a Novel Biodegradable Composite Hydrogel Derived from Gelatin/Chitosan and Polylactic Acid as Slow-Release N Fertilizer

**DOI:** 10.3390/polym15040997

**Published:** 2023-02-17

**Authors:** Weijie Yuan, Sihan Li, Haohui Guan, Shuai Zhang, Yaoxiang Zhang, Min Zhang, Yi Yu, Xiangyi Chen

**Affiliations:** 1 Experimental Center of Forestry in North China, Chinese Academy of Forestry, National Permanent Scientific Research Base for Warm Temperate Zone Forestry of Mountain Jiulong in Beijing, Beijing 102300, China; 2 School of Environmental Science and Engineering, Shaanxi University of Science and Technology, Xi’an 710021, China; 3 Inner Mongolia Imbrication Science & Technology Co., Ltd., Huhhot 010070, China; 4 Beijing King Creation Environmental Protection Technology Co., Ltd., Beijing 102300, China

**Keywords:** polylactic acid, urea, composite hydrogel, slow-release fertilizer, biodegradable

## Abstract

To improve the efficient use of nitrogen and decrease the environmental pollution of N losses, a novel and biodegradable composite hydrogel was prepared by chemical cross-linking synthesis using gelatin (Gel), chitosan (CS) and polylactic acid (PLA) as raw materials. Urea as the nitrogen source was loaded into this new biodegradable hydrogel using the solution immersion method. The chemical structures of the composite hydrogels were characterized and their properties were analyzed by XRD and XPS. The regulation of urea loading and the swelling behavior of the composite hydrogel under different temperature conditions were investigated; the release behavior and release model of the composite hydrogel in the aqueous phase was explored. The results show that the loading of urea is controllable in aqueous urea solution with different concentrations. In the water phase, it shows a three-stage sustained release behavior, that is, the initial release rate of urea is relatively fast, and the medium release rate of urea gradually slows down, and finally the nutrient release rate tends to be flat. The release behavior in the water phase fits to the Ritger–Peppas model. Within 10 min, 180 min and 900 min, the cumulative nutrient release rate of gelatin/chitosan/PLA-urea (GCPU) composite hydrogel is 20%, 70% and 86%, respectively. Compared with pure urea, The urea diffusion time of GCPU was extended by 1350-times. In addition, the GCPU also has good water absorption and water retention properties, in which average water content can reach as high as 4448%. All of the results in this work showed that GCPU hydrogel had good water absorption and retention and N slow-release properties, which are expected to be widely used in sustainable agriculture and forestry, especially in arid and degraded land.

## 1. Introduction

Plant growth and quality are mainly affected by water quantity and fertilizer, and nutrients and drought are the two main factors limiting plant growth, so it is important to improve the use of water and fertilizer nutrients [1]. Urea is the most widely used nitrogen fertilizer [2,3] with high nitrogen content and low production cost, which can be absorbed and used by crops after hydrolysis to ammonium carbonate or ammonium bicarbonate by the action of urease in the soil. However, urea is released faster in the soil [4], and the volatilization and leaching of nutrients cause waste of fertilizer and environmental pollution, especially in regions with soil and water erosion. Therefore, it is a major challenge to improve the effective use of nitrogen, reduce the cost of production inputs, and reduce the environmental impact of nitrogen loss while maintaining plant growth.

The use of highly absorbent composite hydrogels loaded with fertilizers for plant growth and development in this study is one way to reduce water and fertilizer loss [5]. Highly absorbent composite hydrogels were used as controlled release substrates for urea to prepare environmentally friendly slow-release fertilizer hydrogels to reduce the amount and frequency of water use for irrigation and improve soil fertility while promoting plant and crop growth [6]. Hydrogel is a highly absorbent polymer with a three-dimensional mesh structure that can absorb large amounts of liquid, which can reach tens or even hundreds of times more than its own weight, making it a good material for water retention [7]. Slow-release fertilizer hydrogel is a multifunctional material that not only improves the utilization of fertilizer and reduces the environmental hazards caused by fertilizer loss, but also increases the water-holding capacity of the soil due to its good water-holding properties. The raw materials of the slow-release fertilizer hydrogels prepared in this study are gelatin (Gel), chitosan (CS) and polylactic acid (PLA), all of which are biodegradable polymers that are completely converted into products without causing secondary pollution to the environment [8], while the degradation product of PLA, lactic acid, can also be used to improve saline alkaline soils [9].

In our laboratory, the performance of Gel/CS-PLA (GCP) hydrogel for controlled release of water was investigated in the previous work. Based on the previous work, this study further prepared gel/CS/PLA-urea (GCPU) hydrogel slow-release fertilizer, which expands the idea of developing multifunctional materials for promoting plant growth and development by efficiently utilizing nutrients and reducing environmental pollution, especially for vegetation restoration in arid and degraded land.

## 2. Experiments

### 2.1. Raw Materials and Reagents

PLA (Shenzhen Guanghua Weiye Co., Ltd., molecular weight: 100,000, Shenzhen, China); gelatin type A Gel (Tianjin Shengao Chemical Reagent Co., Ltd., strength: 100 g bloom, Tianjin, China); glutaraldehyde (25%d, Shanghai Aladdin Biochemical Technology Co., Ltd., Shanghai, China); span 80 (Tianjin Tianli Chemical Reagent Co., Ltd., Tianjin, China); acetic acid (36% d, analytical reagent. Ltd., Shanghai, China); chitosan (CS, Nippon Soda Trading (Shanghai) Co., Ltd., Shanghai, China, molecular weight: 100,000–150,000 deacetylated >90%), urea (analytical reagent, Tianjin Tianli Chemical Reagent Co., Ltd., Tianjin, China), trichloromethane CHCl_3_ (analytical reagent, Sinopharm Chemical Reagent Co., Ltd.; p-dimethylaminobenzaldehyde (PDAB) (analytical reagent, Tianjin Fuchen Chemical Reagent Co., Ltd., Tianjin, China), H_2_SO_4_ (analytical reagent, Sinopharm Chemical Reagent Co., Ltd., Shanghai, China).

### 2.2. Experimental Instruments and Equipment

Water bath shake (AI-7000-NGD), Shanghai Pudong Physical Optical Instrument Factory (Shanghai, China); X-ray diffractometer (WXRD) (D/Max-3c), Japan Rigalcu Co. (Tokyo, Japan); Ultrasonic cleaning machine (KQ5200DE), Kunshan Ultrasound Instrument Co., Ltd (Kunshan, China); UV-visible spectrophotometer (UV-2600), Unico (Shanghai) Instrument Co., Ltd. (Shanghai, China); Freeze dryer (LGJ-10), Beijing Songyuan Huaxing Technology Development Co., Ltd. (Beijing, China); X-ray photoelectron spectroscopy (Vario EL III), Elementar Analysensysteme GmbH (Hanau, Germany).

### 2.3. Preparation of GCPU Composite Hydrogel

GCP composite hydrogel was prepared by chemical cross-linking method. Firstly, prepare 20 mL of 4% PLA chloroform solution, add surfactant Span 80 for emulsification with mechanical stirring, and set aside of ultrasound for 1 h. Then, 4% gelatin solution was prepared by dissolving deionized water, and 4% chitosan solution was prepared by dissolving acetic acid. Thirdly, gelatin and chitosan solutions were fully mixed with the mass ratio of 1:1 by magnetic stirring for 30 min. Fourthly, 5%,10%,15%,20% and 25% proportions of PLA with emulsification and ultrasound were added, respectively, and continued to be fully mixed; add 2.5% aqueous glutaraldehyde solution; then, freeze-dry the GCP hydrogel product after 6 h cross-linking.

The prepared GCP composite dry gel was immersed in aqueous urea solution with mass fraction of 5–25% for 48 h and fully freeze-dried in a freeze-dryer to obtain the GCPU composite gel loaded with urea.

### 2.4. Representation

WXRD analysis: The composite hydrogel samples were characterized by an X-ray diffraction instrument (D/Max-3c) under the test conditions: Cu target, tube current and tube voltage at 30 mA and 40 kV, scan speed of 5 (°)/min in a scan range of 5 to 80°.

XPS analysis: The binding energy distribution of C, H, O and N elements on the surface of the composite hydrogel material was analyzed using a Vario EL III X-ray photoelectron spectrometer with an X-ray source of Al Kα (hν = 1486.7 eV) at a voltage of 150 W (10 mA, 15 kV) and a surface scan energy range of 0–1200 eV with an accuracy of 1 eV. The surface scan data of the composite hydrogel were processed by Casa XPS software, and the peak separation of different elements was performed.

### 2.5. Performance Test

#### 2.5.1. Water Absorption Performance Test

The equilibrium water absorption properties of hydrogels were determined by the weighing method. The dried hydrogel cylinders of size 12 mm (d) × 12 mm (h) were accurately weighed (*W*_0_) and then completely immersed in deionized water at 25 °C and left to absorb water, and the surface water of the hydrogel was wiped with filter paper dampened with deionized water at intervals and accurately weighed (*W_i_*) and repeatedly measured until water absorption equilibrium. The water absorption *SR* of the hydrogel was calculated as follows (each proportional sample was measured three times in parallel and the results were averaged):

(1) $$SR/\%=\frac{W_{i}-W_{0}}{W_{0}}\times 100\%$$

#### 2.5.2. Water Retention Performance Test

The water retention performance of the hydrogel was determined by the weighing method. The hydrogel with water absorption equilibrium was accurately weighed after wiping the surface moisture with deionized water wet filter paper, which was recorded as *m*_0_. Then, that sample was put into the oven at different temperatures, before taking out and weighing after a certain time, and recording it as *m_i_*. The water retention rate (*WR*%) of the hydrogel sample in the oven was calculated according to the following formula (each proportional sample was measured three times in parallel and the results were averaged):

(2) $$WR\%=\frac{m_{i}}{m_{0}}\times 100\%$$

#### 2.5.3. Urea Loading Rate Test

The prepared GCP composite hydrogels were accurately weighed (*W*_1_) and fully swollen in aqueous urea solution for 48 h hours for urea loading, after which the fully swollen GCPU composite hydrogels were dried for 3 d and weighed accurately (*W*_2_). The urea loading was calculated by (*W*/%) the following equation (each proportional sample was measured three times in parallel and the results were averaged):

(3) $$W/\%=\frac{W_{2}-W_{1}}{W_{1}}\times 100$$

#### 2.5.4. Urea Releasing Test

##### Standard Curve for Sustained Release of Urea

**Figure 1:**
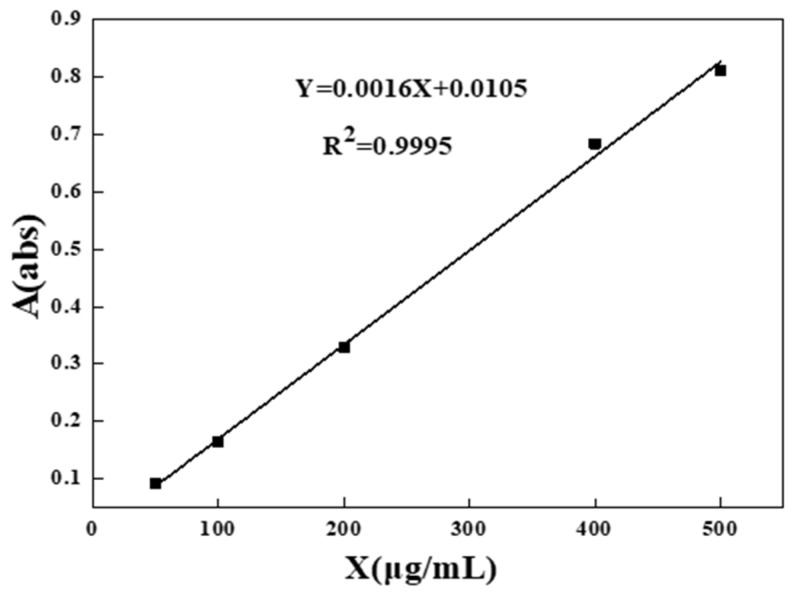
Standard curve of urea.

The urea content in water was measured by colorimetric method using p-dimethylaminobenzaldehyde (PDAB). 10 mL of urea extraction solution (0, 0.5, 1, 2, 4, 5, 8, 10 mL) was taken in a 25 mL colorimetric tube, then 10 mL of PDAB color developer and 4 mL of 2 mol/L H_2_SO_4_ solution were added and, finally, deionized water was added to fix the volume to 25 mL at the scale, mixed and shaken well, and the absorbance was measured at 422 nm after standing for 10 min to confirm the urea content. The absorbance of all solutions was measured and the standard curve was drawn. Linear fitting was made and yielded a correction equation of Y = 0.0016X + 0.0105 (R^2^ = 0.9995), shown in Figure 1.

##### Urea Release Characteristic of GCPU

The prepared GCPU composite hydrogels (12 mm (d) × 12 mm (h)) were immersed in 200 mL deionized water (release medium) without stirring and 1 mL of urea solution was aspirated from the medium at specified time intervals and detected by UV-2600 UV spectrophotometer to calculate the urea concentration and the cumulative release percentage.

To further investigate the release kinetics and the mechanism of urea release from GCPU hydrogels in water, a Ritger–Peppas release model was fitted. Where *M_t_* and *M*_∞_ are the release of accumulated fertilizer nutrients at time t and infinite time, *K* is the release rate constant, *t* is the release time, and *n* is the diffusion index. When *n* ≤ 0.45, the release mechanism is Fick diffusion;; when *n* ≥ 0.89, it is indicated as skeletal dissolution; and when 0.45 < *n* < 0.89 is mixed (dual mechanism) [10].

(4) $$\frac{M_{t}}{M_{\infty }}=Kt^{n}$$

In order to make the data clearer and directly perceived through the senses, the Peppas equation simultaneously was taken as the logarithm to obtain another equation, where lnt was set as *X*-axis, ln(*M_t_*/*M*_∞_) as *Y*-axis, and lnk as the intercept.

(5) $$\ln\frac{M_{t}}{M_{\infty }}=n.\ln t+\ln k$$

To compare with GCPU, the same amount of pure urea was weighed as that in the GCPU hydrogel, and wrapped by etamine in an equal volume of deionized water. After 10, 20, 30, 40 and 50 s, the absorbance value of the immersion solution at 422 nm was measured, and the nitrogen content in the solution and the urea dissolution rate was calculated and obtained.

## 3. Results and Discussion

### 3.1. Structural Analysis (WXRD) of GCPU Composite Hydrogels

**Figure 2:**
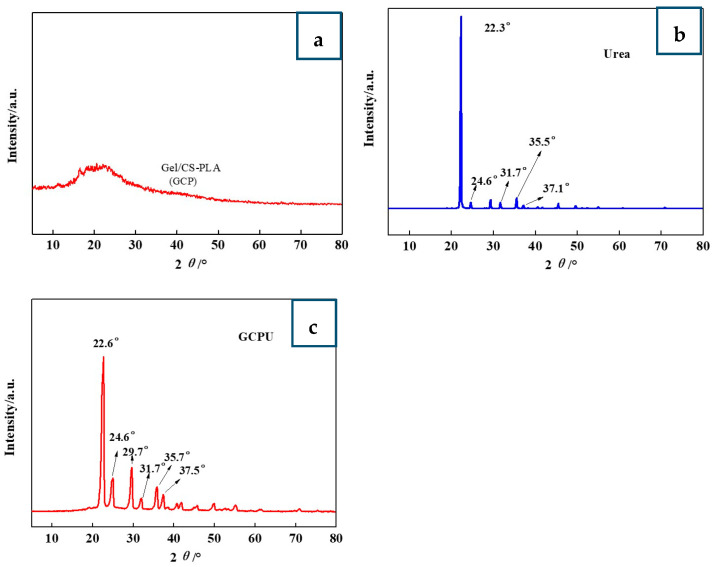
WXRD analysis of GCP (**a**), urea (**b**) and GCPU (**c**).

WXRD profiles of GCPU composite hydrogel are shown in Figure 2. As can be seen from the figure, the characteristic peak of urea of 2 θ = 22.3°, 24.6°, 31.7°, 35.5°, 37.1° indicates that urea has a crystal structure [11]. The characteristic peak of urea appeared in the GCPU composite hydrogel, indicating that urea still has a crystalline structure in the GCP composite hydrogel, and the individual diffraction peaks in the GCPU composite hydrogel due to the macroscopic residual stress, lattice distortion, cell contraction, and the low-angle diffraction peak is slightly shifted to the right. The urea diffraction peak in GCPU was significantly enhanced and the peak width widened, which further indicates that the intermolecular interaction force between urea and hydrogel polymer network, lattice distortion and cell shrinkage may lead to the grain decrease, indicating that urea was successfully loaded in GCP composite hydrogel, and GCPU hydrogel was successfully prepared.

### 3.2. Surface Element Analysis (XPS) of GCPU Composite Hydrogels

**Figure 3:**
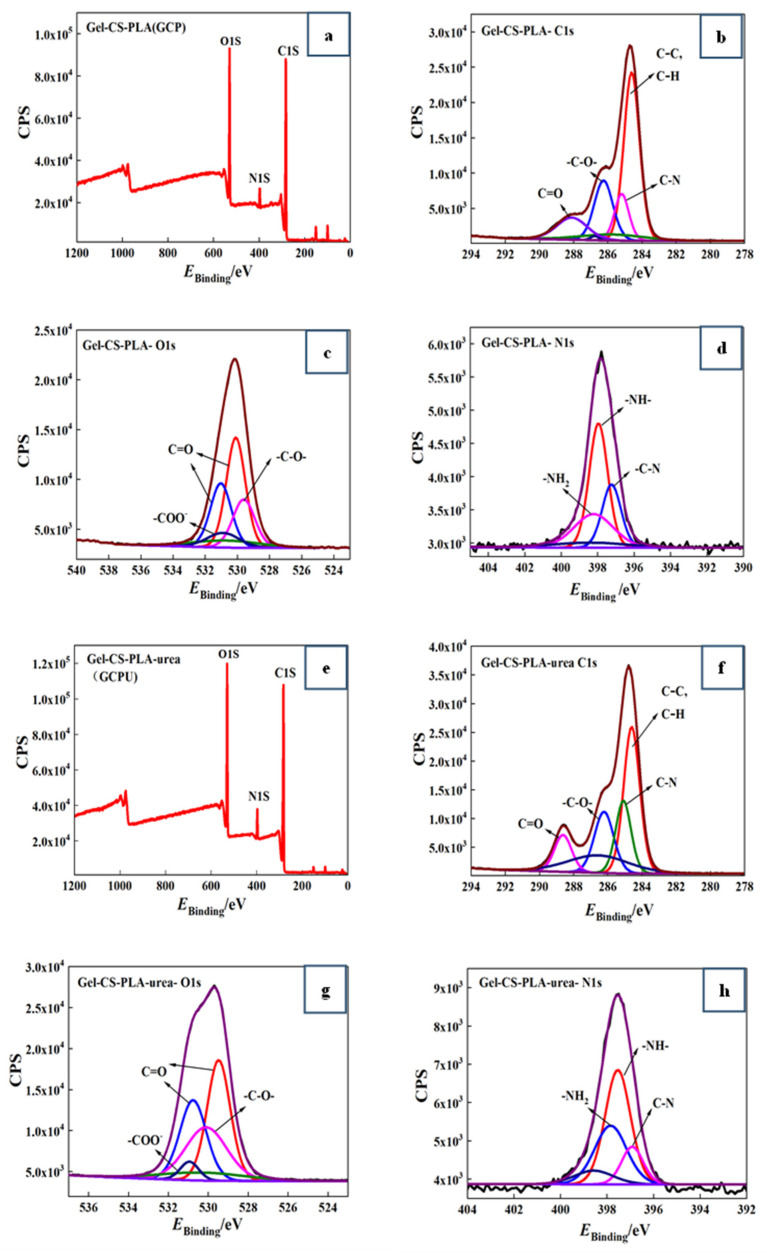
XPS distribution of GCP (**a**) composite hydrogel and the binding energy distribution of C1s (**b**), O1s (**c**), and N1s (**d**) elements; (**e**–**h**) shows XPS distribution of GCPU composite hydrogels and the binding energy distribution of C1s, O1s, and N1s elements.

X-ray photoelectron spectroscopy (XPS) is an advanced analytical technique to study the elemental composition and content, chemical state and chemical bonding information [12]. XPS was used in the study to analyze the chemical bonding state and chemical bonding information of different atoms such as C, O and N. The effect of the addition of urea on the chemical bonding energy of the atoms was explored to study the intermolecular interactions. Figure 3 shows the XPS plots and elemental split peak plots of GCP, GCPU composite hydrogels, respectively, and the chemical bonding energy distributions of C (1 s), O (1 s) and N (1 s) of the composite hydrogel materials are given in Table 1.

It can be seen from the graphs and table that the elemental distribution on the surface of GCP and GCPU composite hydrogels is similar and the elemental count peaks of C1s, O1s and N1s appear. It indicates that the GCP and GCPU composite hydrogels were successfully prepared. The O1s (529.26 ev), N1s (397.82 ev), and C1s (284.45 ev) of GCP are shown in Figure 3a–d, and the O1s (530 ev), N1s (397.82 ev), and C1s (281.94 ev) of GCPU are shown in Figure 3e–h.

GCP and GCPU composite hydrogels appear with five bonding energies at C1s, 284.6 ev, 284.8 ev, 285.1 ev, 286.3 ev, 288.7 ev in the C1 spectral energy of GCPU are C-H, C-C, C-N, C-O, C=O peaks, respectively [13]. N1s of GCPU spectra appearing at 396.9 ev, 397.6 ev, and 397.9 ev as C-N, -NH-, and -NH_2_ peaks, respectively, and the O1s spectra appearing at 529.5 ev and 530.8 ev as C=O peaks, 530.1 ev as C-O peaks, and 530.9 ev as -COO- peaks [14]. The binding energy of the O1s and N1s elemental chemical bonds of GCPU shifted toward lower binding energy compared with GCP. This indicates that non-covalent bonding interactions occurred between polar functional groups in the GCP composite hydrogel after the introduction of urea, and intermolecular interactions such as hydrogen bonding and van der Waals forces between urea molecules and functional groups such as hydroxyl and amino groups in the GCP composite hydrogel, which shifted the chemical bonding energy of elements in the composite.

### 3.3. Urea Loading Rate of GCPU Composite Hydrogels

**Figure 4:**
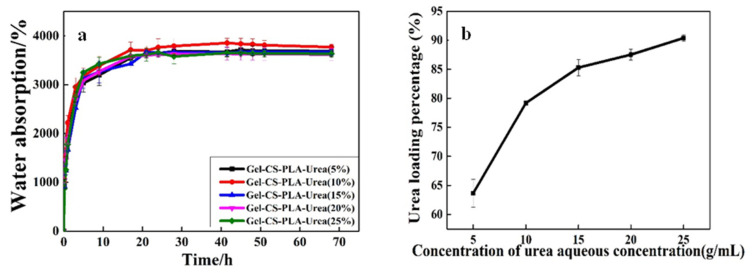
Swelling rate of GCP dry gel in different concentrations of urea aqueous solution (**a**) Urea loading percentage (**b**).

Figure 4 shows the effect of the urea solutions of different concentrations on the urea loading rate of the GCPU composite hydrogel, in which diagram a shows the swelling equilibrium process of urea in different initial urea solution concentrations, and diagram b shows the urea loading rate of the composite hydrogel in the same urea solution concentration.

The GCP composite dry gels were immersed in aqueous urea solutions of different concentrations. The equilibrium swelling was all about 3700% (Figure 4a), but the urea loading percentage increased with the concentration of aqueous urea solution, and the urea loading percentage increased to 90% in the urea solution with a mass fraction of 25% (Figure 4b). The results showed that the equilibrium swelling rates of GCP in aqueous urea solutions with different concentrations were almost equal. However, the urea concentration has a strong effect on the urea loading percentage of the hydrogel. The reason may be that urea is a neutral molecule and no electrostatic repulsion occurs to affect the network of the hydrogel. When the composite hydrogels were immersed in the urea solution, the mechanism of interaction between hydrogel and the solvent will not be changed. Therefore, the equilibrium swelling rate of the composite dry gels can hardly be changed in different concentrations of aqueous urea solutions [13]. However, with the concentration of urea solution increases, there are more urea molecules in the water, which can be absorbed in the network of hydrogel while being immersing in different concentrations of urea solutions. Then, after drying, many more urea molecules remain in the polymer network with higher urea loading percentage and vice versa. This suggests that the urea loading percentage of GCP can be changed by adjusting the concentration of aqueous urea solution to change the urea content in the composite gel [14].

### 3.4. Water Absorption of GCPU Composite Hydrogels

**Figure 5:**
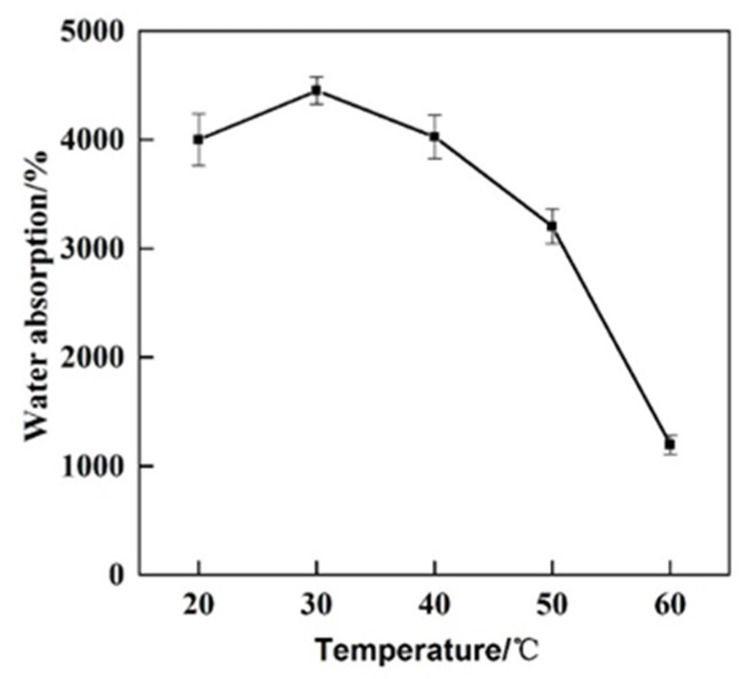
Water absorption of GCPU composite hydrogel at different temperatures.

In order to investigate the water absorption performance of GCPU composite hydrogel under different temperatures, the water absorption performance of GCPU composite hydrogel was tested at 20 °C, 30 °C, 40 °C, 50 °C and 60 °C according to the water absorption performance test method in 2.5.1, and the results are shown in Figure 5.

It is obvious from that, the water absorption capacity of GCPU reached the highest of 4448% at 30 °C. When the external ambient temperature is lower than 40 °C, the equilibrium swelling rate of water absorption of GCPU composite hydrogel does not change much, but with the increase of temperature, the equilibrium swelling rate of GCPU is greatly reduced when it is greater than 40 °C, which may be due to two reasons: (1) The remaining unreacted free end of glutaraldehyde used in the crosslinking process further reacted with gelatine and/or chitosan and consequently increased the crosslinked degree. (2) The gelatine melting point is above 40 °C, so unreacted gelatine may be released in the water medium and, accordingly, the content of hydroxyl groups reduced and, consequently, the amount of water absorption was affected.

### 3.5. Water Retention of GCPU Composite Hydrogels

**Figure 6:**
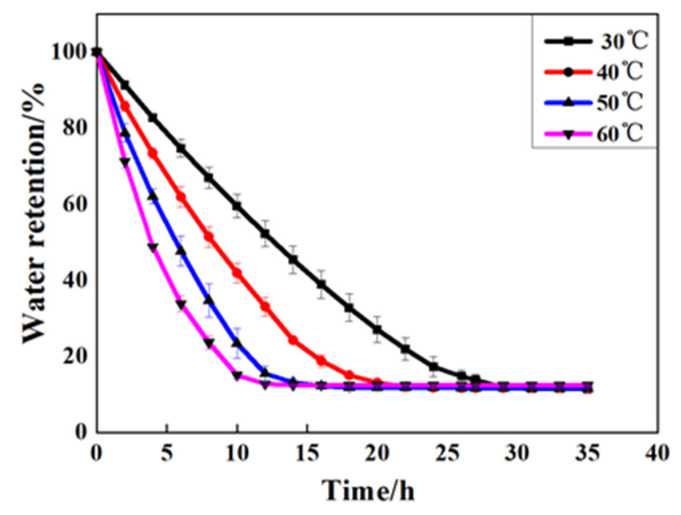
Water retention rate of GCPU composite hydrogel at different temperatures.

In order to investigate the water retention performance of GCPU composite hydrogel under different temperatures, the water retention performance of GCPU composite hydrogel was tested at 30 °C, 40 °C, 50 °C and 60 °C according to the water retention performance test method in Section 2.5.2, and the results are shown in Figure 6.

Figure 6 shows that the water retention rate of the GCPU composite hydrogel could still retain more than 45% after 5 h, and the rate of this material was 80%, 70%, 60%, 45% at 30 °C, 40 °C, 50 °C and 60 °C, respectively, which indicates that the composite gel has good water retention ability even at high temperature. This is because the three-dimensional network of GCPU swelled gradually in the water absorption process, and the hydrophilic group is hydrogen bonded with water molecules, which makes it difficult for water molecules to escape from the gel network structure after water absorption within a certain temperature range. During dehydration, the free water molecules in the structural space of the gel network are the first lost, followed by the bonding water acting with the composite gel through hydrogen bonding. Compared with the bonding water, the free water is more difficult to escape. Most importantly, with the addition of PLA to the GCP, the network structure, rigidity and the control of water molecules of the new composite hydrogel of GCPU were further enhanced, thus the water retention performance of GCPU can be improved. All the results show that the GCPU composite hydrogel has good water retention properties.

Therefore, when GCPU is applied to the soil it can retain moisture under high temperature conditions, effectively reduce irrigation in hot weather and improve soil moisture; in addition, GCPU hydrogel releases nutrients and improves agriculture and forestry yield.

### 3.6. Behavior and Kinetics of Urea Release in Water of GCPU Composite Hydrogels

**Figure 7:**
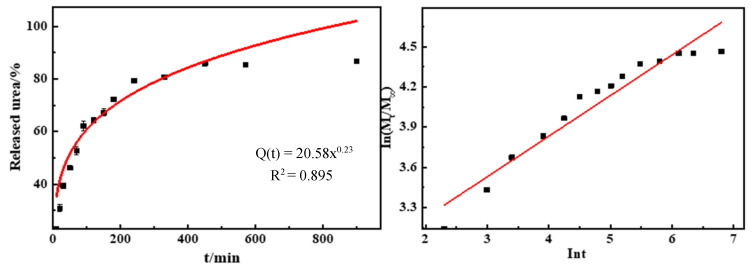
Release rate of GCPU composite hydrogel in aqueous solution.

Figure 7 shows the urea release process and capacity of GCPU hydrogels in water at 25 °C. The cumulative urea release rate of GCPU was 20%, 70% and 86% within 10 min, 180 min and 900 min, respectively, and it can be found that the release of urea exhibits a typical three-stage slow-release behavior [15] with increasing time. The release rate of urea is fast to ensure the timely absorption of plant nutrients in the initial stage, gradually slows down to ensure the uniform diffusion of nutrients in the middle stage, and finally tends to level off to achieve the slow-release effect, which is in line with the growth law of plants and crops. The urea release behavior of GCPU hydrogels could be illustrated as follows: (1) GCPU is transformed into hydrogel by dissolving in water, the concentration of the gel near the interface decreases, a concentration gradient is formed, urea dissolves rapidly and the dissolution rate is larger; (2) when urea passes through the aqueous solution, there are multiple absorption and de-absorption processes between urea molecules and distilled water, the concentration gradient decreases, which helps slow down the release of urea and the dissolution rate gradually decreases; (3) in the late stage of urea release, with the extension of the release time, the release and absorption of urea reach equilibrium, the gradient gradually disappears, the release rate remains stable and finally tends to level off.

From the fitted model showed in Table 2, it can be seen that the release mechanism of urea of GCPU belongs to the Ritger–Peppas release model [16], with n less than 0.45 indicating that the urea molecules undergo diffusive release, and when the GCPU hydrogel dissolves, the network structure increases and the urea molecules dissolve with the immersion of the system solvent, with an R^2^ of 0.925, which has a good correlation, indicating that the Ritger–Peppas release model is applicable to explain the diffusion process [17].

**Figure 8:**
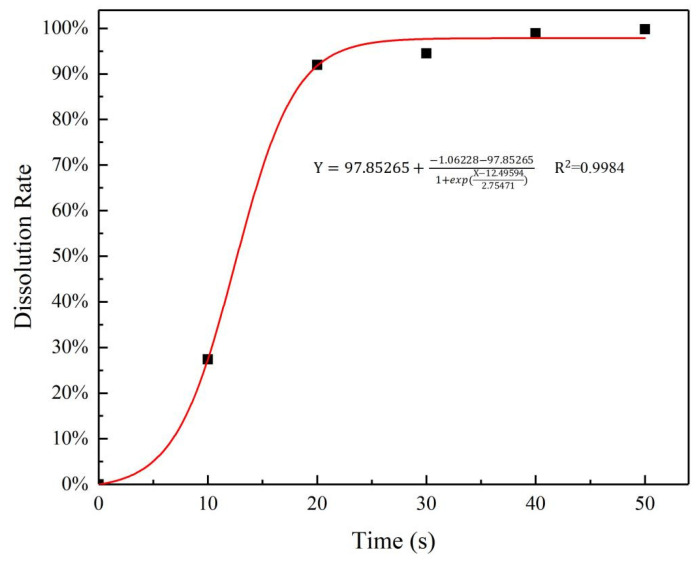
Dissolution rate of pure urea in water.

Nitrogen concentration of pure urea particles dissolved in water at different times is shown in Figure 8. From the figure it can be seen that pure urea particles dissolved quickly in water in the first 20 s, the dissolution rate of urea in the immersion solution increased rapidly, then the increase rate becomes slow, and almost close to 100% after 40 s, which indicates that the urea completely dissolved in water. The comparison between Figure 7 and Figure 8 shows that the urea release rate of the GCPU composite hydrogels is significantly slower, with a diffusion time of about 1350 times longer compared with the pure urea particles.

In conclusion, the GCPU composite material prepared in this study significantly increases the water absorption rate of water-retaining materials, and shows good water retention and fertilizer slow-release performance. Due to the difference between laboratory test conditions and actual field conditions, the nutrient and water absorption characteristics and demands of soil environment and plants in different ecological regions are quite different. Therefore, in order to accelerate the research, development and application of functional fertilizers with both water retention and slow-release characteristics, further study on actual plant growth conditions should be carried out in the future, especially on the appropriate water retention and slow-release amount for mitigating water stress and improving crop nutrient utilization efficiency in arid and degraded land areas.

## 4. Conclusions

An environmentally friendly composite hydrogel was prepared with gelatin, chitosan and polylactic acid, and urea was successfully loaded on gel-CS-PLA hydrogel. The slow-release fertilizer hydrogel has good water absorption and retention properties, and its average water content is up to 4448%; The urea loading percentage of GCPU hydrogel can be adjusted by adjusting the concentration of urea aqueous solution. Urea in water exhibits a typical three-stage sustained release behavior, that is, the release rate of urea in the initial stage is faster, while it is gradually slower in the middle stage, and the final release rate tends to be flat. The release behavior in the water phase fits to the Ritger–Peppas model. Within 10 min, 180 min and 900 min, the cumulative nutrient release rate of GCPU is 20%, 70% and 86%, respectively. Compared with pure urea, the diffusion time of GCPU-loaded urea is 1350 times longer, which indicates that GCPU has a good slow-release effect and can be widely used in agriculture and forestry.

## Figures and Tables

**Table 1:** Binding energy of C 1*s*, O 1*s* and N 1*s* elements in composites.

	C (1 *s*) (eV)				O (1 *s*) (eV)			N (1 *s*) (eV)		
Elemental distribution	C-C C-H	-C-O-	C=O	C-N	-C-O-	C=O	-COO-	-NH-	C-N	-NH_2_
GCP	284.7 284.6	286.2	288.1	285.1	529.6	530.1 531.1	397.9	397.2	398.2	397.9
GCPU	284.6 284.8	286.3	288.7	285.1	529.5	529.5 530.8	397.6	396.9	397.9	397.6

**Table 2:** GCPU Urea In curve release parameters.

Hydrogel	Fitting Model	Slope	Intercept	R^2^
GCPU	Ritger-Peppas	0.303	2.621	0.925

## Data Availability

Data available upon request.
